# Mitochondria: the gatekeepers between metabolism and immunity

**DOI:** 10.3389/fimmu.2024.1334006

**Published:** 2024-02-23

**Authors:** Giovanna Trinchese, Fabiano Cimmino, Angela Catapano, Gina Cavaliere, Maria Pina Mollica

**Affiliations:** ^1^ Department of Biology, University of Naples Federico II, Naples, Italy; ^2^ Department of Pharmaceutical Sciences, University of Perugia, Perugia, Italy; ^3^ Task Force on Microbiome Studies, University of Naples Federico II, Naples, Italy

**Keywords:** immunometabolism, metabolic flexibility, mitochondrial function, mitochondrial dynamics, metabolic reprogramming

## Abstract

Metabolism and immunity are crucial monitors of the whole-body homeodynamics. All cells require energy to perform their basic functions. One of the most important metabolic skills of the cell is the ability to optimally adapt metabolism according to demand or availability, known as metabolic flexibility. The immune cells, first line of host defense that circulate in the body and migrate between tissues, need to function also in environments in which nutrients are not always available. The resilience of immune cells consists precisely in their high adaptive capacity, a challenge that arises especially in the framework of sustained immune responses. Pubmed and Scopus databases were consulted to construct the extensive background explored in this review, from the Kennedy and Lehninger studies on mitochondrial biochemistry of the 1950s to the most recent findings on immunometabolism. In detail, we first focus on how metabolic reconfiguration influences the action steps of the immune system and modulates immune cell fate and function. Then, we highlighted the evidence for considering mitochondria, besides conventional cellular energy suppliers, as the powerhouses of immunometabolism. Finally, we explored the main immunometabolic hubs in the organism emphasizing in them the reciprocal impact between metabolic and immune components in both physiological and pathological conditions.

## Graphical Abstract

**Mitochondria are the scale needle of the immunometabolism.** The metabolic flexibility, mitochondrial damage-associated moleuclar patterns (DAMPs) release, and dynamics are the main processes that modulate the mitochondrial function in response to nutrients and immune signals. Their dynamic balance ensures the body’s immune and metabolic homeostasis.

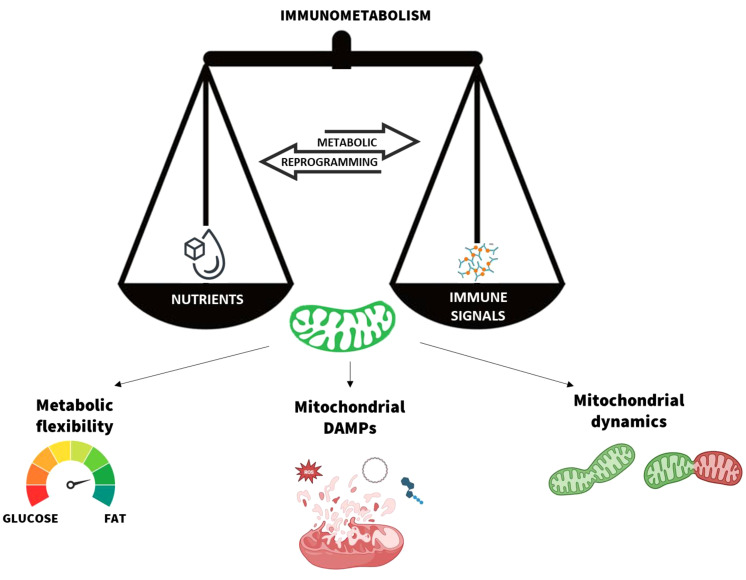

## 1 Introduction

The rules of human survival teach us that: i) we cannot be unarmed against the pathogen attack and ii) nothing survives without nourishment. The “immunometabolism” defines the portal between immunology and metabolism, two trials which our organism trusts to maintain a state of wellness. These processes are inextricably linked and the interfaces between the immune and metabolic systems mediate the whole-body homeodynamics. The crosstalk between these two major balancers of the body health has multiple facets (1). The immune system continually perceives and reacts to pathogenic or environmental dangers with secretion of cytokines, chemokines, and inflammatory mediators by the innate immune cells, and with the proliferation of adaptive immune cells. These processes are bioenergetically expensive and need an accurate control of cellular metabolic pathways (2). The immune response requires the reallocation of nutrients within immune cells in order to: provide the substrates for ATP production serving to sustain the functions of activated immune cells; and build blocks for the production of necessary macromolecules for the proliferation of immune cells. The cellular metabolic reprogramming, that help to regulate specific immune cell functions, is an aspect of immunometabolic research which has already been to some extent explored (3). Indeed, the concept that metabolism influences cellular functions and fate may seem obvious, but taking this step backwards is the appropriate approach for a detailed understanding of the immunometabolic mechanisms, consequently useful to design effective strategies to ensure the health of the organism. The feature of this review is the establishment of a thread which examines the several facets of the immunometabolism, starting from the intersection mechanisms of the mitochondrial metabolism with the functionality of the immune cells. Then, we highlighted the mitochondrial dynamics processes in the activation phases of immune cells, and the immunometabolic regulation in different organs and tissue in both physiological and pathological conditions.

## 2 Metabolic adaptations of immune cells

The cells of the immune system are arguably the most dynamic components of our organism, they need to function in different contexts, including those where the availability of nutrients is restricted or compromised (4). Immune cells possess a broad set of skills ranging from being sleeping sentinels to inducing clonal expansion, modulating surface receptor expression and secreting large amounts of effector molecules (5, 6). The performance of these distinct functional activities is tightly dependent by the metabolic flexibility of these cells (7). Indeed, recent findings have demonstrated that peripheral immune cells can adapt to environmental shifts by metabolizing alternative non- glucose substrates, such as amino acids or fatty acids (8–15). A system in which nutritional and energy inputs are properly processed and substrate utilization is properly regulated is defined as a metabolically sensitive and flexible system. The mitochondrial machinery is responsible for switches in the oxidation of substrates, and the choices are orchestrated by an intricate network of cell signaling events. This metabolic flexibility enables peripheral immune cells to perform a multitude of functions in disrupted environments where the availability of carbon sources varies (16).

In the 20^th^ century, Warburg was the first to launch the immunometabolic research describing the metabolic changes and aerobic glycolysis in cancer cells (17). Recent research has highlighted that each subset of immune cells has a different metabolic control and nutrient utilization. Vats et al. highlighted the molecular pathway that directly links mitochondrial oxidative metabolism to the anti-inflammatory program of macrophage activation, identifying peroxisome proliferator-activated receptor gamma coactivator 1-alpha (PGC1α) and signal transducer and activator of transcription 6 (STAT6) as metabolic regulators that can control macrophage activation (18). To date, increasingly detailed experiments have shown that naïve T and B cells dynamically switch their metabolic programs upon activation. Several studies highlighted signaling and transcriptional networks that regulate metabolic state to tune the management of T cells fate (19). The results converge on the increase of the mitochondrial metabolism and oxidative phosphorylation (OXPHOS) (20–22) and on the induction of anabolic metabolism in T cells, specifically, evidencing that this occurs during the period of quiescence exit in parallel with higher glucose uptake and lactate production (23). Glutaminolysis is an important pathway in these cells, given that glutamine replenishes tricarboxylic acid (TCA) cycle intermediates as they are picked up for biosynthesis (15). These events are mediated by the mechanistic target of rapamycin (mTOR)-associated signaling, in part, by upregulating transcriptional programs mediated by MYC (24), sterol regulatory binding element proteins (SREBPs) (25), and hypoxia inducible factor-1α (HIF-1α) (26–28). T_reg_ cells and memory T cells have a dissimilar metabolism from their activated effector T cells counterparts, indeed, for their development and persistence depend on mitochondrial fatty acid oxidation (FAO) rather than aerobic glycolysis (29).

The interaction between nutrients, metabolic programs and signaling pathways has recently well described also in naïve B cell activation, differentiation and fueling of the antibody secretion machinery. Several studies have found that glucose uptake is increased upon B cells activation (30–33). It was also demonstrated that the initial B cell activation induces metabolic reprogramming, with increased glucose uptake without accumulation of glycolytic metabolites, suggesting that glucose is fluxing through the glycolytic pathway and is probably directed to alternative metabolic pathways in activated B cells. Indeed, stimulated B cells increase programs for OXPHOS, the TCA cycle, and nucleotide biosynthesis (34). The signaling and nutrient sensitive mechanisms that mediate B cell activation and differentiation and the function of energetic and biosynthetic pathways were widely reviewed (35). Several experiments have shown that the functional destination (tolerance, effector or regulatory activities) of B lymphocytes dictates the choice of their metabolic programming, also depending on the receptors and the co-activation molecules stimulated (36). Indeed, glucose restriction did not affect B cell functions, whereas OXPHOS inhibition or glutamine restriction significantly impaired B cell growth and differentiation (34). Metabolic restrictions (such as low ATP reserve and mitochondrial mass, or transcriptional repression of glucose transport and limited activity of the pentose phosphate pathway) provide a safeguard against autoreactive or premalignant B cells (37). This may happen through hyperactivation-induced metabolic stress, such as ATP deprivation and oxidative damage. Meanwhile, prolonged exposure to nutrient oversupply subverts B cell gatekeeper functions, promotes malignant B cell transformation and progression of autoimmune disease (38).

Notable, the metabolic availability influences immune cell fate decisions also through its impact on the epigenome (39, 40). Indeed, several metabolic checkpoints exist to limit epigenetic instability and restrain B cell development (7). Compelling evidence demonstrated that metabolic intermediates are ideal signaling mediators. Their levels are in dynamic equilibrium with systemic, microenvironmental and cell-intrinsic cues, whereby fluctuations inform cell fate decisions (41). They can both inform the fitness of extracellular conditions and integrate this sensing into the epigenome, serving as cofactors for chromatin remodeling enzymes (42). Metabolic-dependent epigenetic reprogramming might explain why changes in cellular metabolism are crucial for multi-stage B cell specification. Lastly, the metabolic state influences immune cell function not only through epigenetic remodeling but also through a restructuration of intracellular architecture, this will be discussed in more detail below in a dedicated section.

## 3 Immunometabolic skills of the mitochondria

In recent years, the scientific research has produced new striking knowledge of mitochondrial function in metabolism, leading to consider the mitochondria as targets for the development of new therapeutic approaches. Alongside the paradigm widely described in biochemistry textbooks, which defines these organelles as ‘energy powerhouses’ of the cell, a new image has emerged of the mitochondria as a ‘Pandora’s box’, an intracellular ‘container’ crucial not only for the life but also for the cell death (43, 44). For this reason, it is essential to consider their expertise in immunometabolic management.

Mitochondria represent the most ancient endomembrane system in eukaryotic cells. They arose around two thousand million years ago and, over the years, mitochondria have continuously demonstrated their autonomy and ability (45). In 1907, they were defined as cellular organelles responsible for the functions of respiration and energy production (46). Around the 1950s, Kennedy and Lehninger discovered that the TCA, FAO and OXPHOS take place in the mitochondria (47). In 1967, Margulis revived the long-forgotten endosymbiont theory on the origin of organelles (48). Since the 1970s, the mechanism of mitochondrial biogenesis has been elucidated and it is recognized that mitochondria are semi-autonomous organelles, capable of synthesizing 5% of the proteins they require and importing the rest from cytoplasmic synthesis (49). Morphologically, like their bacterial ancestor, mitochondria consist of two separate and functionally distinct outer and inner membranes that enclose the intermembrane space and matrix compartments. The architecture of mitochondria is essential for their proper functioning and also for the containment of immunogenic molecules derived from mitochondria (50, 51). They also contain the mitochondrial DNA (mtDNA), a circular genome, which has been reduced in the course of evolution through gene transfer to the nucleus. The bacterial-like characteristics of mitochondria also reinforce the idea that they are hubs of immunity (52). The proteins present in mitochondria are structurally similar to those in bacteria and allow them to be recognized by the same receptors as the immune system (53). To date, as already hypothesized by Altmann in 1890, the main role of mitochondria is to provide metabolic energy in all eukaryotic cells (54). However, these organelles orchestrate mechanisms which directly impact cell fate and fitness, so to consider them trivially ‘powerhouses of cells’ would be limiting, to say the least. Indeed, it is well known that the metabolic functions of mitochondria reach far beyond bioenergetics. Additionally to their exclusive ability to carry out the OXPHOS, these organelles participate in intermediary metabolism, regulate programmed cell death, calcium homeostasis, and control the production of reactive oxygen species (ROS) (55–57).

A proper mitochondrial functionality is fundamental for the cellular homeostasis. In this regard, several molecules extruded from mitochondria alert neighboring cells, the immune system (58), and the producing cell itself about mitochondrial dysfunction (59). Several studies demonstrated that mitochondrial ROS also contribute to adaptive stress signaling pathways, such as hypoxia and control cell proliferation and differentiation (60, 61). Likewise, the levels of nitric oxide (NO), another by-products of mitochondrial respiratory activity, act as initiators through which mitochondria modulate signal transduction pathways implicated in the induction of cellular defense mechanisms and adaptive responses (62). Mitochondria are also the source of molecules, including proteins, lipids, metabolites and mtDNA, collectively named damage-associated molecular patterns (DAMPs). These DAMPs are endogenous danger molecules that are released from damaged or dying cells and activate the innate immune system by interacting with pattern recognition receptors. The DAMPs, when imbalanced, employ immunogenic capacity in immune and non-immune cells (63). ATP, succinate, cardiolipin, N-formyl peptides (NFPs), mtDNA and mitochondrial transcription factor A (TFAM), are examples of DAMPs that serve as danger flags for immunological signaling (63). The secretion of succinate triggers pro-inflammatory differentiation of T-lymphocytes (64) and have synergic effects with Toll-like receptors ligands in dendritic cells for the production of cytokines. The succinate is a regulator of inflammation, in M1 macrophages due to a break point of Krebs cycle it was observed its accumulation, and demonstrated a prominent proinflammatory activity and roles in immunity (65). High levels of extracellular ATP signals induces release of pro-inflammatory cytokines, inflammasome activation (66), neutrophils degranulation, apoptosis and ROS release through P2X receptors (67, 68). The exposition of cardiolipin to the extracellular media is associated with increased apoptosis and autophagy: the cardiolipin can bind directly to Nod-like receptor 3 (NLRP3) and activate inflammasome-mediated immune response (69). Moreover, cardiolipin externalization to the outer mitochondrial membrane acts as an elimination signal for mitophagy in mammalian cells (70), this process is facilitated by the activated Gasdermin D permeabilization of mitochondrial membranes that cause rapid, cardiolipin-dependent mitochondrial destruction (71). N-formyl peptides are extruded by the mitochondria of damaged or dying cells, they act as chemoattractant of neutrophils via formyl-peptides receptors (72). Extra mitochondrial mtDNA has been widely shown to induce a proinflammatory state (73, 74), its binding to TLR9 induces proinflammatory cytokines production, chemotaxis and phagocytic activation via a MyD88-dependent signaling cascade (75). TFAM enhances the immunogenicity of mtDNA (76), TFAM is recognized by the receptor for advanced glycation end products, which guides TFAM-mtDNA complexes to the endosomal pathway, also, TFAM enhances cytokine secretion in combination with NFPs (77). mtDNA may also amplify the activation of NLRP3 by mitochondrial ROS (mtROS) (78). ROS are a consequence of mitochondrial disruption in both M1 macrophages (40) and effector T cells and control adaptive immune-cell activation. It is noteworthy that ROS are also guiding signals in the production of inflammatory cytokines. T and B cells require ROS production to trigger an adequate immune response. T cell activation induces a spike in mtROS production, and blockade this process neutralizes IL-2 production by T cells (79). B cell activation is also managed by mtROS. The activation of B cell-surface receptors stimulates in turn the calcium release into the cytoplasm, which promotes ROS production, this cooperative interaction acts in a feedback manner to amplify the early signal generated (80). An interesting research showed that isolated human monocytes exposure to mtDAMPs generated significantly less interleukins IL-1β, IL-6, IL-12-p70 and tumor necrosis factor-α (TNF-α) upon lipopolysaccharide challenge when compared to their untreated counterparts, leading to speculate to the induction of a transient state in which these cells are refractory to further endotoxin stimulation (81). Further studies will be crucial to understanding the role of this phenomenon, that could be the root of the onset of noncommunicable chronic diseases, typified by mitochondrial dysfunction and disruption of the immune system. Recently, the intermediate role of mitochondria in toll-like receptor-mediated innate immune responses and in the activation of the NLRP3 inflammasome complex has highlighted, supporting the striking functions of mitochondria in innate immunity (82). Indeed, besides being DAMPs producers, mitochondria are also linked to immunity through their role as innate immune platforms that host the mitochondrial antiviral signaling protein (MAVS) as a viral RNA sensor and the inflammasome NLRP3 as a multiple immunogenic receptor (63).

Understanding how non-immune cells respond to DAMPs released following mitochondrial harm and the mechanisms implicated in these responses are among the main targets of recent researches (63). Understanding the conditions under which damage to non-immune cells leads to chronic and systemic inflammatory responses is relevant. This is discussed in a later section.

## 4 Mitochondrial dynamics drive the immunometabolic pathways

The multifaceted contributions of mitochondria to cell metabolism as bioenergetic powerhouses, biosynthetic centers, ROS production managers and waste management hubs is undisputed (83). Unequivocally, mitochondria perform a plethora of cellular functions besides energy production (84). It is equally clear that the fate and function of innate and adaptive immune cells depends crucially on mitochondrial bioenergetics (58). Exciting evidence demonstrated that mitochondria constantly change their morphology depending on the cell’s metabolic requirements, highlighting reciprocal crosstalk between mitochondrial dynamics and metabolism (85, 86).

Mitochondrial dynamics refers to the formation of a dynamic network involving a continuous alternation of fusion and fission/division processes in order to maintain their cellular abundance, morphology, quality and function control (87). Mitochondrial fusion and division typically counterbalance each other. Three proteins that control mitochondrial fusion and division have identified: i) mitofusins (MFNs) (outer mitochondrial membrane fusion), ii) Optic-atrophy-1 (OPA1)/mitochondria genome maintenance 1 (inner mitochondrial membrane fusion), and iii) Dynamin-related protein-1 (DRP1)/Dynamin-1 (division of outer and inner mitochondrial membranes) (88). Mitochondrial fusion is the physical merging of the mitochondrial membranes of two originally distinct mitochondria. Mitochondrial division is the separation of a single organelle into two or more independent structures. These two active and combined effects originate the mitochondrial networks. Several studies performed in metabolic tissues, such as the liver, the skeletal muscle and the central nervous system demonstrated that the unbalance in mitochondrial fusion/fission dynamics cause cell and tissue dysfunction and altered metabolic homeodynamics (89–92). A decade ago, it was already proven that deletion of any of the dynamics machinery perturbs OXPHOS and glycolytic rates at baseline (93). Tissue-specific deletion of mitofusin-2 (Mfn2) in muscles of mice disrupts glucose homeostasis (94), and Drp1 ablation in the liver results in reduced adiposity and elevated whole-body energy expenditure, protecting mice from diet-induced obesity (95). The evidence that the alterations in fusion/fission machinery alter the mitochondrial function and with it the cell function holds true across various tissues, including the immune system. Indeed, mitochondrial dynamics are a critical control point also for immune cell function (96).

In several immune cells, including neutrophils, macrophages, mast cells, and T- and B-cells, mitochondria adapt specific mitochondrial morphologies according to the cellular activation state (78, 97, 98). In LPS-activated macrophages the inhibition of mitochondrial fission, through the Drp1 inhibitor Mdivi-1, reduce glycolytic reprogramming that these cells implement to achieve polarization into a proinflammatory M1 state (97). The mitochondrial dynamics impact also on the role of the mitochondrial membrane as a signaling platform. Indeed, the depletion of Mfn1/2 or Opa1 reduced MAVS-driven innate antiviral signaling in a mitochondrial membrane potential-dependent manner (99, 100). In neutrophils, the mitochondrial fusion is implicated in the formation of neutrophil extracellular traps (NET), the deletion of OPA lead to a decrease of ATP levels which is fundamental for microtubule network assembly and NET formation (101). Also in human mast cell immune response was investigated the role of mitochondrial dynamics revealing that degranulation processes and secretion of preformed TNF are regulated by Drp1 activation (98). The different roles of T- lymphocytes as T effectors (T_e_) and T memory (T_m_) cells impose them large changes in ATP demand and nutrient utilization. T_e_ cells promote aerobic glycolysis to sustain anabolic pathways of metabolism, while T_m_ cells engage catabolic pathways, like FAO, and these metabolic differences are reflected in mitochondrial morphology (96). Memory T cells have more fused mitochondrial networks suggesting a requirement for mitochondrial fusion in memory T cell metabolism and homeostasis. Indeed, the fusion protein OPA1 is required for tight cristae organization in T_m_ cells, facilitating efficient electron transport chain (ETC) activity and favorable redox balance, its deletion caused defects in T_m_ survival (97). While, more fragmented mitochondria (fission process, with low expression of the fusion proteins MFN2 and OPA1 and high levels of active DRP1) were observed in T_e_ cells, leading to punctate mitochondria, cristae expansion and reduced ETC efficiency which promote aerobic glycolysis (97, 102). In T cells the processes of IL-2 production and immune synapse formation are dependent on mitochondrial fission (103, 104). Indeed, activated T cells show an increase in the production of mtROS, required in the activation of the transcription factor NF-kB, which transcribes IL-2. Inhibition of DRP1 by Mdivi-1 reduced IL-2 mRNA levels and T-cell proliferation (96).

Mitochondrial fission also occurs during B-cell activation, while naïve B cells have predominantly elongated mitochondria. Activated B cells increase glucose uptake, TCA cycle and OXPHOS and have fragmented mitochondria, while naϊve B cells maintain a predominance of elongated mitochondria (30, 34). These findings are similar to those noted in T cells; however, it was found that naϊve B cells have significantly fewer mitochondria in comparison to naϊve T cells (96, 105). It seems that B cells use the mitochondrial remodeling as a key mechanism to control the optimal function of these few mitochondria to compensate this restriction in the number and volume of mitochondria and ATP reserves (106). B cells predominantly favor mitochondria fission and thus house smaller, less functional mitochondria with limited capacity for oxygen consumption and ATP production (34, 107). However, a recent study demonstrated that T cell–dependent activation of murine B cells not only temporarily increased metabolic activity (e.g., glucose uptake and glutamine consumption) but also increased mitochondria number through fission in the absence of mtDNA replication (34).

These exciting evidence highlight that mitochondria are tightly interlaced with metabolic and immune cell homeostasis, it follows that the proper function of mitochondria is crucial to ensuring the health of the organism. From now on, talking about mitochondrial-driven immunometabolic homeostasis would not be a hazard.

## 5 Immunometabolism in health and disease: the main immunometabolic hubs in the organism

**Figure 1:**
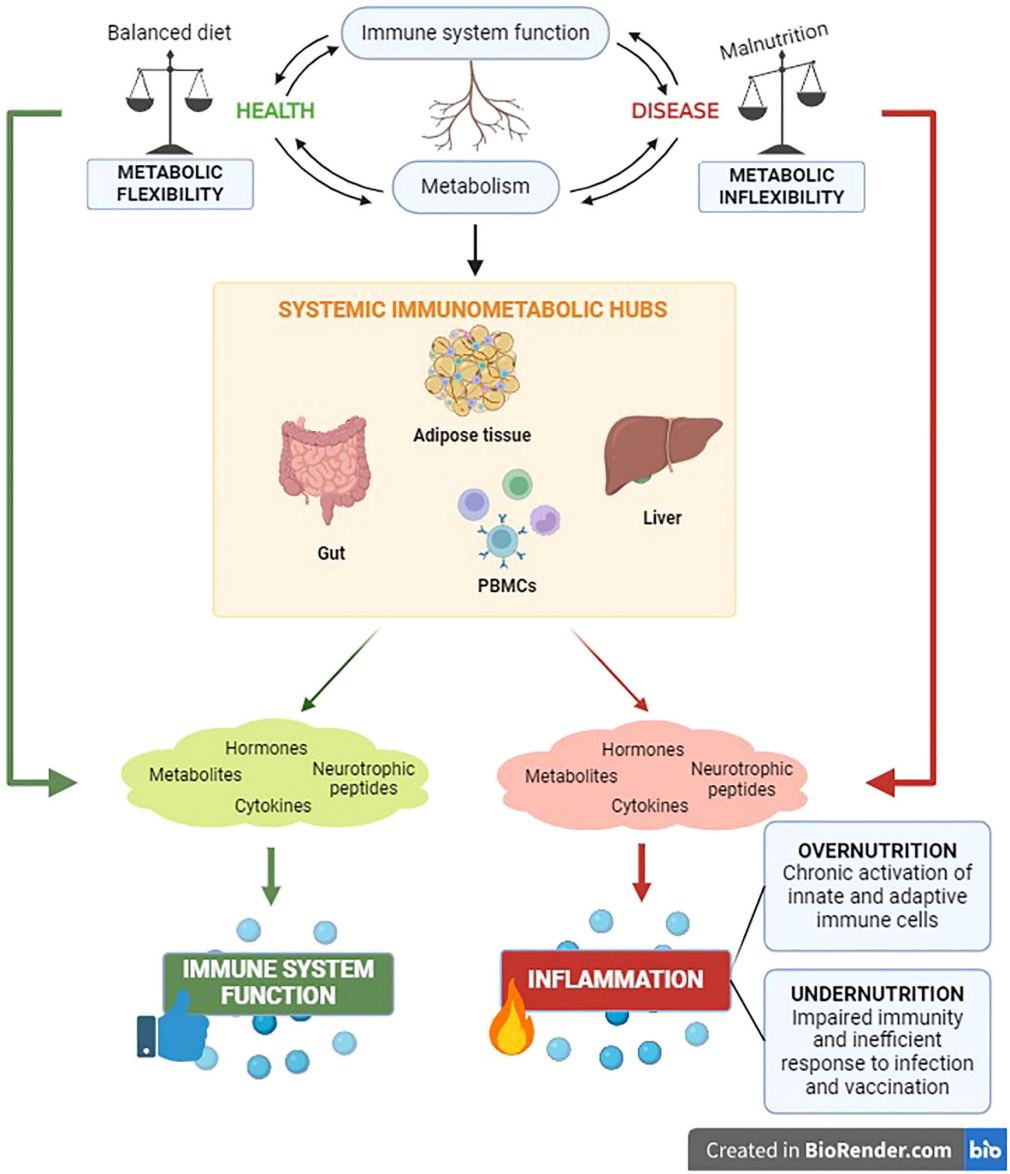
The immunometabolic control has significant clinical relevance for the health status of the organism. The reciprocal impact between metabolic and immune components unveils in the main body’s immunometabolic hubs in both physiological and pathological conditions.

Metabolic homeostasis and immune function are pivotal requirements at the root of systemic health monitoring. The crosstalk between immune and metabolic processes is coordinated by communication circuits between specialized tissue-resident cells and organs that include messenger molecules such as hormones, neurotrophic peptides, cytokines, and metabolites (108–112). The intervention of these components of systemic immunometabolism underlie the impact of the metabolism on systemic inflammation and vice versa, both in health and in disease (**Figure 1**).

Nutritional habits are key determinants of body composition and systemic metabolism (113). Meantime, it is well known that malnutrition leads to inflammation and influences systemic immune responses (114, 115). The unbalance between the body energetic management (metabolism) and the ability to defend itself against pathogens (immune response) has critical implications for the occurrence of a wide range of chronic noncommunicable diseases, including obesity, diabetes, cardiovascular pathologies and cancer (108). Indeed, it has been well established that chronic inflammation is the trigger of the above-mentioned diseases (108) and, recently, the immunological adaptations in response to nutritional status have been highlighted. Undernutrition impairs immunity, causing inefficient responses to infections and vaccinations. Conversely, the metabolic overload in obesity can affect immunometabolism favoring chronic activation of both innate and adaptive immune cells, with subsequent low-grade systemic inflammation and altered susceptibility to autoimmune diseases (116). A thorough understanding of the intracellular network and intercellular correlations that regulate immunometabolism systemically is quite complex. For this reason, below we attempt to trace the main immunometabolic hubs of the organism.

### 5.1 Adipose tissue

The adipose tissue was identified as an important immune cell niche during homeostasis and an important immune-metabolic communication hub in metabolic syndrome (108, 117–121). Indeed, a wide range of immune cells are accumulated in the adipose tissue in the course of diet induced-obesity influencing the systemic metabolism (122, 123). The macrophages accumulate within adipose tissue produce the inflammatory cytokines IL-6 and TNF-α (124, 125). Locally, IL-6 can induce lipolysis in neighboring adipocytes and impair lipoprotein lipase, decreasing adipocyte lipid depot. The high circulating levels of IL-6 and free fatty acids (FFAs) promote insulin resistance and immune cell function alterations affecting both local and distal tissue microenvironments. Moreover, the accumulation of FFAs promotes ROS production in macrophages, which in turn augments activation of the NLRP3-ASC inflammasome (126), and peripheral insulin resistance, mediated by the secretion of the IL-1β. When inflammation persists, like in obesity and in non-communicable diseases, prolonged disruption of metabolic homeostasis could lead to immune cell dysfunction and dysregulated systemic metabolism. In this context, we are faced with ‘sterile metabolic inflammation’ that consists of persistent inflammation in the absence of infection (127). Here, the bilateral crosstalk between aberrant metabolism and immune regulation is disrupted and results not only in disease progression but leads also to immune senescence (128). Buck et al. have extensively reviewed the metabolic instruction of immunity and attempted to shed light on how feeding behaviors can also affect host immune fitness, but the relationship between the variety of factors that influence the systemic metabolism and immune cell activity is only beginning to be explored (7).

Recently, several lines of evidence on why the metabolic overload from obesity blunt the immune system and increases the vulnerability to infectious and autoimmune diseases have been reported (116, 129). Firstly, a functional impairment of both the innate and adaptive immune system is attributable to chronic low-grade inflammation that lead to impaired chemotaxis, dysregulated production of pro- and anti-inflammatory factors and altered macrophages differentiation (130). This disrupts the delicate balance of adipose tissue between its function of immunologically active adipocytokines-producing organ, and the action of the latter on affecting adipocyte homeostasis and metabolism (131, 132). Moreover, the altered immunometabolism in obesity could lead to autoimmunity. The immune cell differentiation may be impaired in obese people due to excessive stimulation of nutrient- and energy-sensing pathways (such as increased mTOR activity) in immune cells, with consequent increase of proinflammatory TH1 and TH17 cells and decreases Tregs, which increase the risk of self-tolerance lack (116). Lastly, exciting research demonstrated that the increased adiposity observed in obese people increase also the bone marrow adiposity (133), resulting quickly in a profound hematopoietic insult with reduction of lymphocyte population and compromised immune function (134–136).

### 5.2 Gut

The gut can be considered the main immunometabolic interface of the organism representing a barrier surface, where a single layer of epithelial cells is the main mediator of crosstalk between gut microbes in the lumen and host cells, including immune cells in the lamina propria (DCs, macrophages, innate lymphoid cells, and T cells). Peyer’s patches are scattered along the epithelium which house germinal centers that maturate IgA-secreting B cells with the help of follicular helper T-cell help. B cells augment glycolysis upon activation and depend on pyruvate import for longevity as long-lived plasma cells (7). The epithelial cells and intraepithelial lymphocytes in the gut coordinate the tightly regulated immune responses, implemented for both avoiding detrimental responses to commensals or food antigens and adequately respond to pathogens (137). It is noteworthy the role played by the short-chain fatty acids (SCFAs), metabolites produced by commensal bacteria able to influence B cell metabolism and boost antibody responses in both mouse and human B cells, promoting Ig A secretion (138). The presence of SCFAs and vitamins support the maintenance of barrier function by promoting the development and survival of Tregs and innate lymphoid cells. Noteworthy, homeostatic immune-driven signals secreted by gut resident immune cells (e.g., IL-10) may also mediate their effects through metabolic modulation (7). Indeed, it was demonstrated that the deficiency of the pleotropic anti-inflammatory cytokine IL-10 in macrophages is sufficient to recapitulate the onset of severe colitis in mice (139). Possibly, the metabolic shift towards to aerobic glycolysis during innate immune cell activation is the explanation of the anti-inflammatory activity of the IL-10.

### 5.3 Liver

The liver can be defined as the immunometabolic controller of the organism. The central role played by the liver in the immune-metabolic homeostasis being well recognized (140). The function of the liver as the main metabolic organ inevitably exposes it to newly produced neo-antigens, enhancing the risk of overactivation of components of the immune system with potentially harmful consequences for hepatic cell homeostasis (141). Several evidence point out the importance of the liver as “regulatory system” where different immune and non-immune cell populations work together in order to protect the host from antigenic overload of dietary components and drugs derived from the gut, facilitating tolerance rather than immunoreactivity (141, 142). Indeed, the immune cells coexist in a close symbiotic manner to support the hepatic metabolic functions (143). In the liver, naïve T cells recirculating within the sinusoids make direct contact with sinusoidal cells, such as liver sinusoidal endothelial cells (LSECs) or Kupffer cells (144). Gut-derived food antigens are picked up by Kupffer cells, LSECs, and liver dendritic cells and presented to naïve T cells, leading to immune tolerance of both CD8+ T cells and CD4+ T cells (145). In addition, compelling evidence demonstrated that virus-induced innate immune responses in hepatocytes are mediated by the antiviral cytokine type I interferon (IFN-I) that apart from inducing an antiviral state, rewires cellular metabolism of innate immune cells to boost the production of immune-modulatory metabolites (146–148) and modulates cellular redox homeostasis and central metabolic pathways in hepatocytes (113, 149–151). Moreover, the portal blood delivers to the liver numerous factors derived from gut and visceral adipose tissue (e.g. pro-inflammatory cytokines, lipids and bacteria-derived factors, such as endotoxins) (152) that seem to be critical in the systemic and central inflammation (153). These endocrine and immune mediators, in turn, can modulate the hepatic metabolism by influencing the bioenergetic regulation of hepatic mitochondria. Conversely, inflammation-induced metabolic reprogramming of hepatocytes can influence systemic energy metabolism (154). These data highlight the important role of the liver as central modulator of systemic immunometabolism and strengthen the bidirectional cause-effect relationship between mitochondrial metabolic stress and immune regulation.

### 5.4 Peripheral blood mononuclear cells

PBMCs are immunometabolic sentinels of the organism. PBMCs are circulating cells able to sense and respond to systemic metabolic and inflammatory stressors. They circulate continuously throughout the body in the bloodstream, and are subject to changes in blood composition, including those related to fluctuations in nutrients, substrates and hormones (155, 156). PBMCs can be defined sentinel cells able to respond either to internal signals (such as hormones) or external ones (such as nutrients) and to reflect energy metabolism of internal tissues with which they interact, as well as their gene expression profile. In addition, these cells contain respiring mitochondria and, therefore, are a functional biomarker in translational bioenergetics (157). For this reason, PBMCs represent a suitable system to study changes in cell metabolism and to control the management of immune surveillance (156). Moreover, since several researches in animal models demonstrated that PBMCs can reflect the metabolic framework that cannot or can hardly be sampled in humans, such as liver and brain (158–160). These cells can be used as a surrogate tissue to monitor nutritional responses and provide predictive disease risk markers (161).

## 6 Discussion and conclusions

This review has highlighted that immunometabolic control has significant clinical relevance to the health status of the organism. Furthermore, it has left no doubt that mitochondria are the main players in this fine-tuning between metabolism and immune function, finding the metabolic flexibility of immune cells and the mitochondrial dynamics processes to be the secrets of appropriate immunometabolic homeostasis (see also graphical abstract). In particular, we underlined the importance of the metabolic flexibility of immune cells, exploring the role of their metabolic pathways and how this regulates the outcome of the immune response. On the one hand, it is well established that initial T and B cells activation, during the period of quiescence exit, lead to increased glucose uptake and promote aerobic glycolysis (23). Then, effector T and B cell subsets display differences in metabolic activities on the basis of their subsequent functional specialization (23). T_reg_ cells and memory T cells revert to a catabolic state and rely mainly on mitochondrial fatty acid oxidation (162). Also metabolic programs of activated B lymphocytes change depending on their functional destination (tolerance, effector or regulatory activities) and on the receptors and co-activation molecules stimulated (36). In addition, we explored the ability of mitochondrial DAMPs to employ the immunogenic capacity in immune and non-immune cells, and the significant relevance of mitochondrial ROS production in the trigger of an adequate immune response (163, 164). We traced the thread that leads from malnutrition to metabolic inflexibility in immune and non-immune cells, with consequent systemic meta-inflammation and disruption of the immune system. At once, a proper metabolic regulation supports immune cell activities in physiological contexts, while dysregulated immunometabolism contributes to pathophysiology. In the last chapter of the review, we explored and discussed the intricate intracellular networks and intercellular correlations in the main immunometabolic hubs of the organism. We highlighted as the interference of mitochondrial dysfunction (unbalanced DAMPs and ROS production and metabolic inflexibility) influence also non-immune components and lead to chronic and systemic inflammatory responses, typical features of non-communicable diseases.

For this reason, it is advisable to pursue a constant and in-depth exploration of immunometabolism, both in the detailed molecular pathways involved and with an interdisciplinary approach. The aim is to identify mitochondrial targets useful for the development of new intervention therapies that could help reduce the global burden of metabolic, inflammatory and autoimmune diseases.

## Author contributions

GT: Conceptualization, Investigation, Visualization, Writing – original draft, Writing – review & editing. FC: Investigation, Writing – review & editing, Writing – original draft, Visualization. AC: Writing – original draft. GC: Writing – original draft. MM: Conceptualization, Project administration, Validation, Writing – review & editing.
